# Ovarian Vein Thrombosis Can Be a Complication of Hysteroscopy

**DOI:** 10.7759/cureus.16196

**Published:** 2021-07-05

**Authors:** Aneesh Kumar, Sorab Gupta

**Affiliations:** 1 Internal Medicine, Bronx Care Hospital Center, New York City, USA; 2 Hematology and Oncology, Bronx Care Hospital Center, Bronx, USA

**Keywords:** ovarian vein thrombosis, venous thromboembolism, pregnancy, pulmonary embolism, abdominal pain

## Abstract

Ovarian vein thrombosis is not well understood, and there is no consensus regarding treatment. It can present with subtle symptoms and is not usually high on the list of differentials. Traditionally, most cases are linked to pregnancy and postpartum state, but our case adds to the growing list of non-puerperal patients diagnosed with ovarian vein thrombosis after an outpatient procedure. In an era where there is a drive for minimally invasive procedures and shorter hospital stays, there is a need to have specific guidelines to direct the diagnosis and treatment of this rare form of thromboembolism.

## Introduction

An ovarian vein thrombosis (OVT) is a rare form of venous thromboembolism, which occurs in 0.02% to 0.18% of all pregnancies [1]. Although most cases occur in puerperal patients, it has been diagnosed in non-puerperal conditions like pelvic inflammatory diseases, endometritis, malignancy, sepsis, thrombophilia, pelvic surgeries, and gynecologic procedures [2]. As is the case of our patient, even fewer cases have been associated with minimally invasive pelvic procedures like a hysteroscopy [3]. We report the case of a 39-year-old nonpregnant female who developed OVT after hysteroscopy for a malpositioned intrauterine device and was successfully treated with a direct anticoagulant.

## Case presentation

A 39-year-old gravida 5 para 3 female was admitted for sharp, right lower quadrant, non-radiating, abdominal pain, which started three weeks prior and was progressively worsening to the point where it had become unbearable. She denied abdominal distension, nausea, vomiting, diarrhea, fever, chills, vaginal discharge, polyuria, dysuria, and hematuria. She reported that she had a progesterone intrauterine device for three years, which was removed uncomplicated four weeks prior under ultrasound guidance due to malposition. She had a history of two first trimester miscarriages. She had no previous history of blood clots and no family history of thromboembolic or bleeding disorders.

She was afebrile and normotensive at the presentation. Her abdominal examination revealed a soft and diffusely tender abdominal wall, with deep tenderness in the right lower quadrant. No guarding or rigidity was noted, no palpable organs, and there were no signs of free fluid. A pelvic exam performed by the gynecology team revealed normal external genitalia, pink cervix, anteverted uterus with no palpable adnexal masses. Her urine pregnancy test was negative. The initial laboratory values showed a white blood cell count of 8.1 (4.8-10.8 k cells/µL), platelets 379 (150-400 k cells/µL), hemoglobin 10.7 (12.0-16.0 g/dL), serum lipase 27 (≤61 U/L), prothrombin time 12.5 (9.9-13.3 seconds), and partial thromboplastin time 37.9 (27.2-39.6 seconds). Her reverse transcription-polymerase chain reaction (RT-PCR) for SARS Coronavirus was negative.

**Figure 1:**
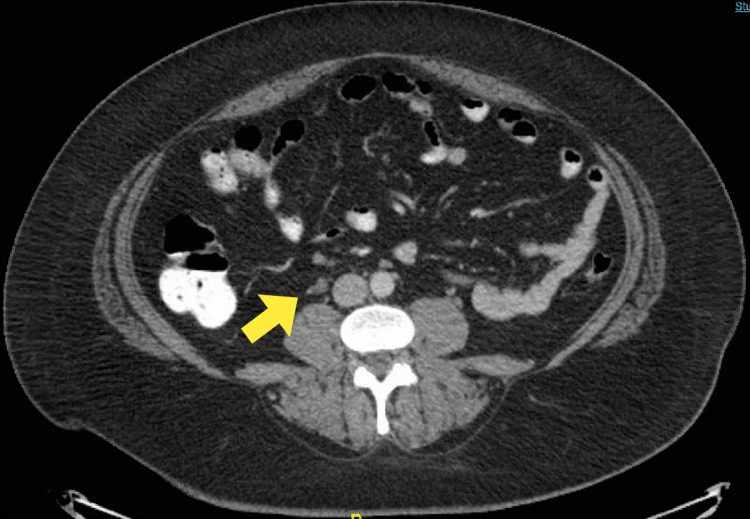
Computerized tomography of abdomen showing enlarged and thrombosed right ovarian vein

The computerized tomography imaging of her abdomen and pelvis with intravenous and oral contrast revealed an enlarged and thrombosed right gonadal vein (Figure 1). No other pathology was identified. A hypercoagulable workup was requested due to the spontaneous nature of the thrombus - factor V Leiden mutation analysis, prothrombin gene G20210A mutation, antiphospholipid antibody panel, antinuclear antibody screen, autoantibodies to proteinase-3 (PR-3), and myeloperoxidase (MPO) were negative. Protein C functional assay was 141% (reference 70%-180%), protein S functional assay was 79% (60%-140%), and antithrombin III assay was 102% (80%-135%). She was treated with apixaban 10 milligram twice a day for a week, followed by 5 milligram twice a day, and was seen in the hematology clinic three weeks later. She reported significant improvement in her symptoms and continues to take apixaban, with the plan to discontinue treatment after three months, after repeat imaging.

## Discussion

OVT was first described in 1956 [4]. The true incidence and prevalence are not clear. In peripartum patients, OVT occurs more commonly on the right side (60.0%, P = 0.03), but overall incidence is similar on both sides (44.6% right side and 41.4% left side) [5]. The predilection to involve the right ovarian vein can be explained by its acute angle of entry into the inferior vena cava, compression by a dextrorotated uterus, antegrade blood flow in the right ovarian vein, longer length, and the absence of competent valves [6,7]. The pathogenic factors include hormonal changes, compression of the inferior vena cava, an imbalance between coagulation and fibrinolysis and slowing of blood flow after delivery [8].

The diagnosis of OVT is a clinical challenge. The most common symptoms reported are fever (80%), abdominal pain(55%), malaise, nausea, vomiting, and rarely, shortness of breath, and a palpable intra-abdominal mass. Our patient did not experience a fever at any point, and abdominal pain had a waxing-waning course. There are a myriad of conditions that can present similarly, including ovarian torsion, tubo-ovarian abscess, pelvic inflammatory disease, puerperal endometritis, appendicitis, inflammatory bowel disease and pyelonephritis [6-8]. The definitive diagnosis required imaging of the ovarian veins. Color Doppler ultrasonography is the most cost-effective, easily available, and safest option. Computerized tomography imaging is the most rapid. Magnetic resonance angiogram has a sensitivity and specificity of almost 100% [9]. The complications of OVT are potentially fatal. Pulmonary embolism can occur in 3%-33% of cases, with mortality up to 4%. OVT can also extend into the inferior vena cava and renal veins. Other rare complications reported are ovarian infarction, ureter obstruction, and kidney rupture [4]. In one study, thrombosis recurred in 9.9% of women, was remote from the original OVT, and was associated with greater mortality [10].

Anticoagulation forms the crux of treatment, however, the agent of choice, dose, and duration of treatment is debatable. Duration of treatment ranges from three to six months among authors and lifelong treatment for hypercoagulable diseases [7]. While some studies advocate the use of Vitamin K antagonists [5], others prefer low molecular weight heparin [11], although one study has demonstrated clot resolution with rivaroxaban [12]. One study showed that the treatment guidelines for lower extremity thrombosis could be extrapolated to OVT [13], and hence, our patient was prescribed apixaban for treatment. In patients who present with fever, we can use beta-lactam with beta-lactamase inhibitors, third-generation cephalosporins, metronidazole, clindamycin or gentamicin, for a period of 2-7 days based on clinical response [11]. We also want to highlight other reports where OVT has been linked to COVID-19 infection [14]. With the ongoing pandemic, the number of such cases is only expected to rise, and OVT should be a part of the differential.

## Conclusions

OVT is now being increasingly recognized as a cause of vague abdominal symptoms in puerperal and nonpuerperal females. A high index of suspicion is required to facilitate prompt diagnosis, early treatment and to help reduce morbidity and mortality due to dreadful complications. There is a need for further prospective studies to establish guidelines regarding management.
